# A 7‐year‐old boy with toxic epidermal necrolysis, heart failure, and sepsis treated with the guidance of invasive hemodynamic monitoring: A case report

**DOI:** 10.1002/ccr3.4430

**Published:** 2021-07-06

**Authors:** Amir Saeed, Nima Mehdizadegan

**Affiliations:** ^1^ Department of Pediatrics Division of Intensive Care Unit Shiraz University of Medical Sciences Shiraz Iran; ^2^ Cardiovascular Research Center Shiraz University of Medical Sciences Shiraz Iran

**Keywords:** case report, heart failure, invasive hemodynamic monitoring, sepsis, skin, Toxic epidermal necrolysis

## Abstract

Toxic epidermal necrolysis is a rare immunological disease that is secondary to some medications or upper respiratory infections, with more than 30% involvement of skin and mucosa. Herein, we describe a 7‐year‐old boy with TEN, heart failure, and sepsis treated with the guidance of an invasive hemodynamic monitoring device.

## Graphical abstract

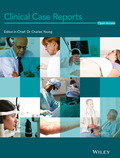

## 1 INTRODUCTION

Toxic epidermal necrolysis is a rare immunological disease that is secondary to some medications or upper respiratory infections, with more than 30% involvement of skin and mucosa. Herein, we describe a 7‐year‐old boy with TEN, heart failure, and sepsis treated with the guidance of an invasive hemodynamic monitoring device.

Toxic epidermal necrolysis (TEN) and Steven‐Johnson syndrome (SJS) are rare immunological diseases in the skin that are secondary to some medications (the most common cause) such as sulpha drugs, phenobarbital, NSAIDs, or upper respiratory infections. They are presented with skin and mucosal involvement and differentiated according to the percentage of body surface area is involved; less than 10% in SJS, more than 30% is defined TEN, and 10%‐30% is the overlap of these two.^1^

TEN is commonly present with fever and flu‐like symptoms and then skin and mucosal involvement are seen. The incidences of TEN were reported 0.4 cases per million children per year in the United States.^2^

Although the mortality rate of TEN is lower than adult patients,^3^, ^4^ these patients are prone to multiple organ failure and infection due to extensive skin involvement; so they should be treated like burn patients and in cases with extensive skin involvement, they need to be treated in an intensive care unit (ICU).

Here, we describe a 7‐year‐old boy that presented with TEN following to viral infection (Chickenpox) that was complicated with infection and treated with the guidance of Pulse Contour Cardiac Output (PiCCO) and survived without any sequelae.

## 2 CASE PRESENTATION

**FIGURE 1:**
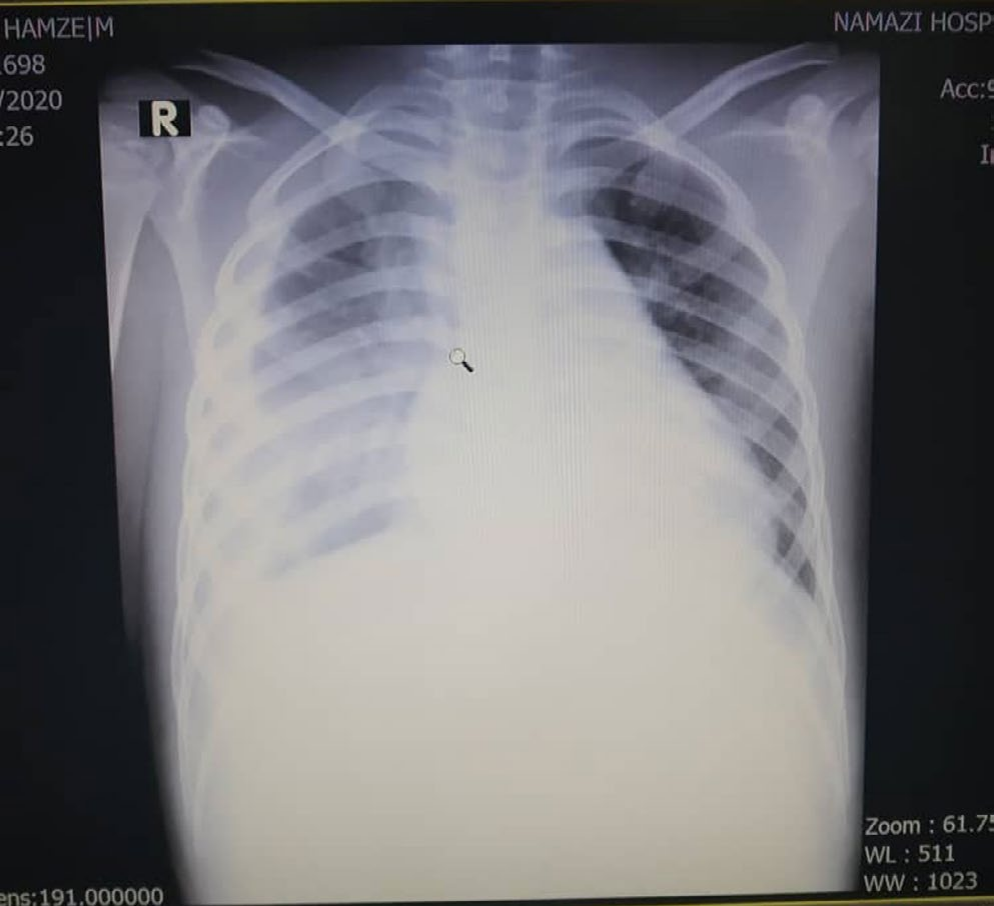
The first chest X‐ray

**FIGURE 2:**
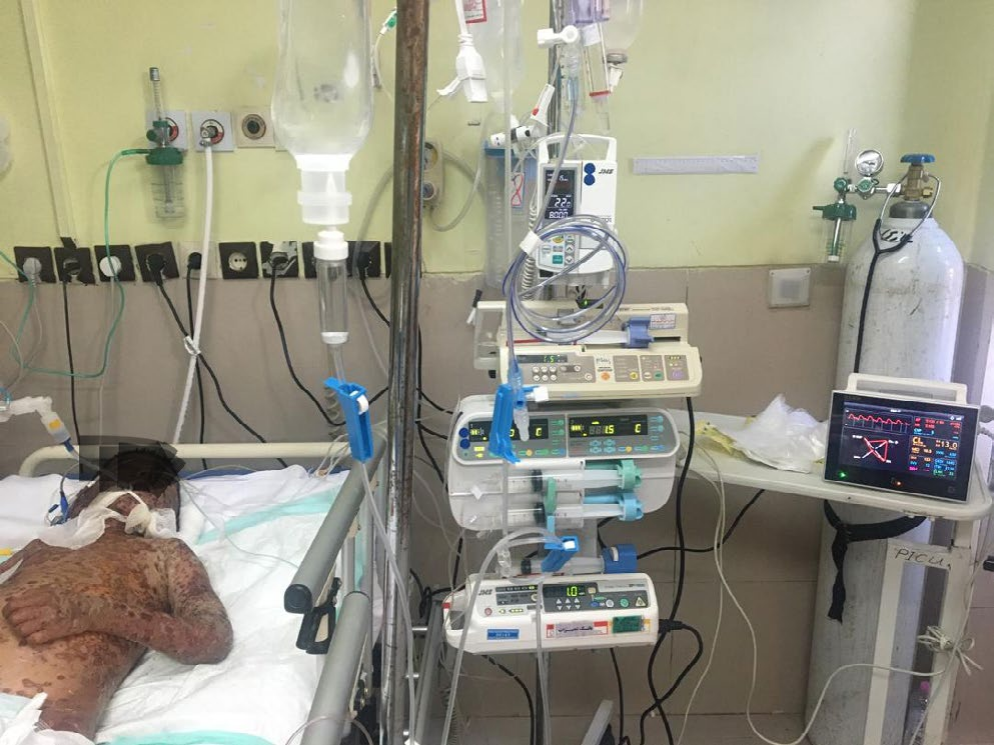
The first day of PICU admission

A patient was a 7‐year‐old boy that presented with fever and following fever developed vesicles and blisters on body, visited by a pediatrician in his town and with the diagnosis of chickenpox got some emulsion for skin and antihistamine for itching, but after 2 days his conditions deteriorated to extensive involvement of skin and mucosa, so admitted in his town's hospital. On the 3rd day, due to worsening of his condition, transferred to the pediatric intensive unit (PICU) of Namazi Hospital (Namazi Hospital is a main tertiary referral center located in Shiraz city in the south of Iran). on arrival to PICU, his vital signs were as follows: Glasgow coma score(GCS):13/15, blood pressure: 120/80, heart rate: 145, temperature: 36.5, respiratory rate: 42, and arterial blood gas: PH: 7.20, PCO2: 20, PO2: 52, HCO3: 11, base excess:‐18, O2 saturation:86% (Figures 1,2). In physical examination, capillary refill time was more than three seconds, redness and detachment of the top (epidermal) layer of the skin in the whole body (>90% BSA), involvement of mucosa(oral and genitalia) and ocular involvement, Nikolsky's sign was also positive; so with the diagnosis of TEN+sepsis; admitted and broad‐spectrum antibiotic, intravenous immunoglobulin (IVIG) started and because of refractory decreased O2 saturation, he was intubated and mechanical ventilation started (Figures 1,2).

For continuous hemodynamic monitoring, an arterial line inserted in femoral artery and a central venous catheter was inserted in the subclavian vein connected to an invasive monitoring device; PiCCO (Pulse Contour Cardiac Output). PiCCO is one of invasive hemodynamic monitoring that uses a combination of two techniques for advanced hemodynamic and volumetric monitoring: transpulmonary thermodilution for volumetric measurements of preload and cardiac output and Pulse contour analysis to provide continuous cardiac output and stroke volume variation (cardiac index (CI), systemic vascular resistance (SVR), mean arterial pressure (MAP), pulse pressure variation (PPV), and stroke volume variation (SVV)).^5^

**FIGURE 3:**
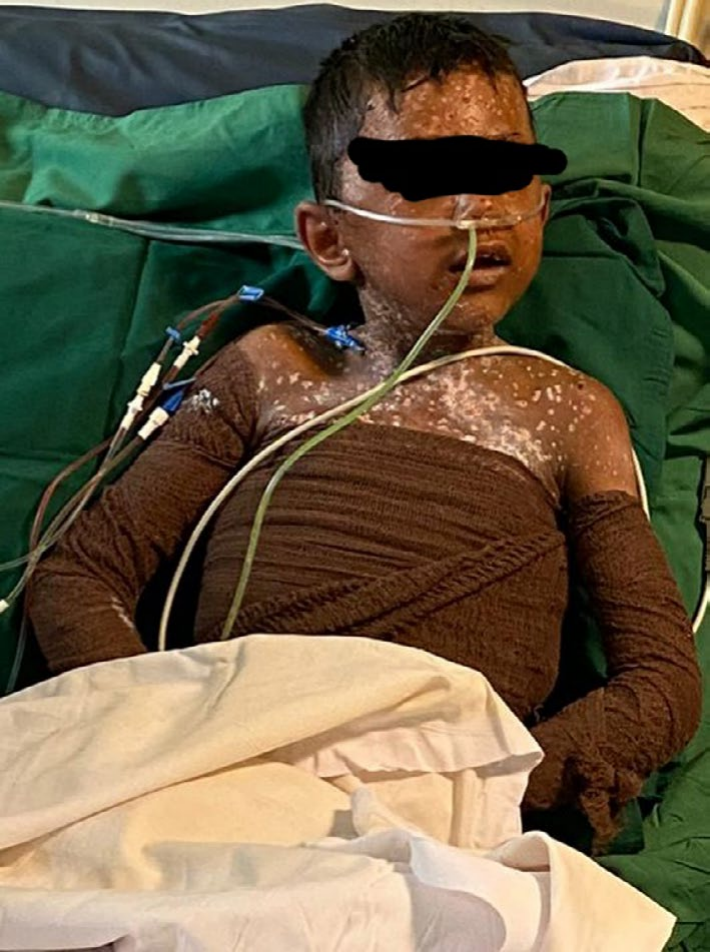
The last day of PICU admission

The initial hemodynamic variables and laboratory data were as noted in Table 1 and 2. Echocardiogram report was as follows: dilated right atrium and right ventricle, poor right ventricular systolic function. According to hemodynamic data, laboratory findings, and clinical evaluation, the patient management proceeded. During the PICU admission, the patient had frequent daily dressing for skin and eye care. After 9 days, the patient was extubated, o2 was delivered via nonrebreathing mask, and 16 days later discharged to home with good condition and without any sequella (Figure 3).

**TABLE 1:** Hemodynamic parameters

	1st day	6th day
Heart rate	145	92
CVP +((mm Hg)	23	10
SCVO2	90%	80
Sys BP(mm Hg)	123	124
Dia BP(mm Hg)	84	74
MAP(mm Hg)	97	91
CI(L/min/m2)	12.6	3.64
ITBI	1556	462
ELWI (cc/kg) (3‐7)	19	8
GEDI (cc/m2) (680‐800)	1245	580
SVRI (1700‐2400)	472	1724
PPV (%)(0‐10)	9	9
SSV (%)(0‐10)	11	12
PVPI (1‐3)	1.6	2.1

Abbreviations: CI, cardiac index; CVP, central venous pressure; dBP, diastolic blood pressure; EVLWI, extravascular lung water index; GEDI, Global end‐diastolic index; ITBI, intrathoracic blood volume index; MAP, mean arterial blood pressure; PPV, pulse pressure variation; PVPI, Pulmonary vascular permeability index; SBP, systolic blood pressure; SCVO2, central venous o2 saturation; SVRI, β systemic vascular resistance index; SVV, stoke volume variation.

**TABLE 2:** Laboratory findings

Laboratory data (normal range)	Result
White blood cells (count/mL)	25 900
platelet	63 000
Procalcitonin ≤0.3	8.8
C‐ reactive protein <6 (mg/L)	136
Creatine phosphokinase (U/L) M: <171 F:<145	1620
Lactate dehydrogenase (U/L) <480	3250
Aspartate transaminase (U/L) M: < 37 F: < 31	71
Alanine aminotransferase (U/L) M: <41 F: <31	84
Albumin	2.4
Blood urea nitrogen (mg/dL) 8 ‐ 20	79
Creatinine M: 0.8‐1.3 F: 0.6‐1.2	0.7
Pt /INR	18.9/1.96
Skin culture	Candida nonalbicans
Blood culture	pseudomonas
ESR	84
Serum glucose	380
Serum bicarbonate	11
Sodium	153
Potassium	7
Chloride	111
Total bilirubin	6.1
Direct bilirubin	3.8

## 3 DISCUSSION

Wound care, fluid and electrolyte management, eye care, nutritional support, and temperature control are the mainstay of treatment of TEN.

There are some criteria for predicting prognosis such as increased blood urea nitrogen (BUN) and serum creatinine level, respiratory failure, and sepsis, but the Score of Toxic Epidermal Necrosis (SCORTEN) is a good tool for prediction of mortality in TEN; although at first it was used in adult patients, some studies showed its validity in pediatric patients^6^; our patient's SCORTEN was 5 that was equal to about 90% mortality, but our patient had some additional risk factors such as respiratory failure, infection, and heart failure that are not listed in SCORTEN, but hemodynamic parameter‐guided therapy, nutritional support, and infection control help us to cure this patient.

Fluid loss in TEN is estimated one‐third of patients with burn,^7^ but the estimation of the amount of fluid to resuscitate has been always challenging; we can use clinical and laboratory data such as heart rate, blood pressure, urine out, or hemodynamic monitoring.^8^ In our patient, we used clinical and laboratory data in addition to hemodynamic parameters to guide us for hydration and according to parameters of volume, he did not need fluid as hydration.

Our patient was in high cardiac output failure (high cardiac output state is defined as cardiac index of greater than 4.0/min/m^2^ and decreased systemic vascular resistance index [SVRI]),^9^ according to hemodynamic parameters; the treatment focused on fluid restriction, treatment of sepsis, monitoring for volume overload, and frequent usage of diuretics (the parameters of 6th day’s improvement were impressive [Table 1]).

## 4 CONCLUSION

Although it is recommended for aggressive fluid treatment in patients with TEN, in severe cases; the amount of fluid can not only be evaluated with clinical data and advanced hemodynamic monitoring is needed to guide the treatment.

## CONFLICT OF INTEREST

None declared.

## AUTHOR CONTRIBUTIONS

AS: planned the study and wrote the manuscript and submitted the manuscript. NM: gathered patients' data and edited the manuscript. Both authors contributed to the final manuscript.

## ETHICAL APPROVAL

This study was approved by the ethics committee of Shiraz University of Medical sciences with approval ID: IR.sums.med.rec.1398.133. Written informed consent was obtained from the parents and sent to the ethics committee.

## CONSENT FOR PUBLICATION

Obtained.

## DATA AVAILABILITY STATEMENT

The data that support this case report are available from the author upon reasonable request.
